# Exploration of serum biomarkers for predicting the response to Inchinkoto (ICKT), a Japanese traditional herbal medicine

**DOI:** 10.1007/s11306-017-1292-x

**Published:** 2017-11-08

**Authors:** Masahito Uji, Yukihiro Yokoyama, Katsuya Ohbuchi, Kazuaki Tsuchiya, Chiharu Sadakane, Chika Shimobori, Masahiro Yamamoto, Masato Nagino

**Affiliations:** 1 Division of Surgical Oncology, Department of Surgery, Nagoya University Graduate School of Medicine, 65 Tsurumai-cho, Showa-ku, Nagoya, Japan; 2 Tsumura Research Laboratories, Tsumura & Co., Ami, Japan

**Keywords:** Metabolomics, Biliary obstruction, Choleretic effect, Herbal medicine

## Abstract

**Introduction:**

In patients with obstructive jaundice, biliary drainage sometimes fails to result in improvement. A pharmaceutical-grade choleretic herbal medicine, Inchinkoto (ICKT), has been proposed to exert auxiliary effects on biliary drainage; however, its effects are variable among patients.

**Objectives:**

The aim of this study is to explore serum biomarkers that are associated with pharmaceutical efficacy of ICKT.

**Methods:**

Obstructive jaundice patients who underwent external biliary decompression were enrolled (n = 37). ICKT was given orally 3 times a day at daily dose of 7.5 g. Serum and bile samples were collected before, 3 h after, and 24 h after ICKT administration. The concentrations of total bilirubin, direct bilirubin, and total bile acid in bile specimens were measured. Metabolites in serum samples were comprehensively profiled using LC–MS/MS and GC–MS/MS. Pharmacokinetic analysis of major ICKT components was also performed.

**Results:**

ICKT administration significantly decreased serum ALT and increased bile volume after 24 h. The serum concentrations of ICKT components were not well correlated with the efficacy of ICKT. However, the ratio of 2-hydroxyisobutyric acid to arachidonic acid and the ratio of glutaric acid to niacinamide, exhibited good performance as biomarkers for the efficacy of ICKT on bile flow and ALT, respectively. Additionally, comprehensive correlation analysis revealed that serum glucuronic acid was highly correlated with serum total bilirubin, suggesting that this metabolite may be deeply involved in the pathogenesis of jaundice.

**Conclusions:**

The present study indicates that ICKT is efficacious and provides candidates for predicting ICKT efficacy. Further validation studies are warranted.

**Electronic supplementary material:**

The online version of this article (10.1007/s11306-017-1292-x) contains supplementary material, which is available to authorized users.

## Introduction

Despite improvements in surgical techniques and refinements in pre- and postoperative care, hepatectomy in patients with obstructive jaundice is still associated with high morbidity and mortality rates (Yokoyama et al. 2016). Preoperative biliary drainage has been performed to improve outcomes after surgery via improvement of liver function (Kawashima et al. 2013). However, the excretion of biliary substances such as bilirubin and bile salts is sometimes insufficient, presumably due to the impairment of intrahepatic bile formation and transport. The elucidation of mechanisms and cellular components involved in such impairment, therefore, will facilitate the development of a novel efficient therapy for hepatectomy in obstructive jaundice patients.

Inchinkoto (ICKT) is a pharmaceutical-grade Japanese traditional herbal medicine having potent anti-apoptotic (Yamamoto et al. 2000) and choleretic (Shoda et al. 2004) effects. The drug has been reported to exert prominent beneficial effects in severe hepatitis, biliary atresia, and primary biliary cirrhosis patients (Yamamoto and Shoda 2005). ICKT is composed of three crude medicinal plants, *Artemisiae Capillari Spica, Gardeniae Fructus*, and *Rhei Rhizom*a. Genipin, the bacterial metabolite of the major compound geniposide in *Gardeniae Fructus*, has been reported to have various hepatoprotective and choleretic properties including the promotion of MRP2-mediated anion excretion into the bile (Amamoto et al. 1996; Yamamoto et al. 2000; Leu et al. 2003; Shoda et al. 2004; Okada et al. 2007). 6,7-dimethylesculetin, another major active component present in *Artemisia Capillaris Spica*, activates constitutive androstane receptor, which is a key regulator of bilirubin clearance in the liver (Huang et al. 2004). These properties suggest possible beneficial effects of ICKT on biliary excretion in obstructive jaundice patients with biliary drainage. However, the effect of ICKT has been found to vary among individuals.

Regarding geniposide and 6,7-dimethylesculetin, it has been reported that they are absorbed systemically in the case of Yin Chen Hao Tang, which is composed of the same medicinal plants as ICKT (Wang et al. 2011). However, genipin was not measured in these studies. Since genipin is thought to be an essential component to exert the benefit of ICKT, the ability to convert from geniposide to genipin might be associated with the effectiveness of ICKT. Therefore, we attempted to measure the concentration of genipin in the blood after ICKT administration and evaluate the correlation between the effect of ICKT and genipin concentration in the blood. Additionally, potential biomarkers may exist that are capable of distinguishing ICKT responders and non-responders. In this study, we performed serum metabolomics to explore biomarkers for predicting the efficacy of ICKT.

Metabolomics is increasingly being applied to various clinical conditions (Nishiumi et al. 2012; Lau et al. 2015a, b). Because metabolomics is located downstream of other omics, such as genomics, transcriptomics, and proteomics, it is expected that it would provide valuable information which is more closely related to the phenotype. Therefore, metabolomics may be a useful tool to facilitate the identification of the pathophysiological conditions of obstructive jaundice and pharmacological mechanisms of ICKT, as well as biomarkers for diagnosis and prediction of disease states and therapeutic efficacy.

In this study, we administered ICKT in patients with biliary drainage due to obstructive jaundice. Blood samples were collected before and after ICKT treatment, and sera were subjected to metabolome analysis to obtain a better understanding of the disease state and responsiveness to ICKT treatment in each patient. The concentrations of ICKT components, such as genipin, geniposide, and 6,7-dimethylesculetin, were also determined. We then explored candidate biomarkers useful for predicting ICKT efficacy and providing information about the pathogenesis of jaundice.

## Materials and methods

### Patient recruitment

From August 2014 to March 2016, patients with obstructive jaundice who underwent external biliary decompression either by endoscopic naso-biliary drainage (ENBD) or percutaneous transhepatic biliary drainage (PTBD) were enrolled in this study. Informed consent was obtained from all patients. Patients who refused to participate in this study and who were taking other choleretic drugs (such as ursodeoxycholic acid) were excluded. The study protocol was approved by Nagoya University Ethics Committee and registered in the University Hospital Medical Information Network (ID, UMIN000020274; http://www.umin.ac.jp/).

### Administration of ICKT

Two to four days after biliary decompression, ICKT (TJ-135; Tsumura & CO, Tokyo, Japan) was given orally three times a day at daily dose of 7.5 g (2.5 g per packet). This dosage is used in clinical practice and has been approved by Ministry of Health, Labor and Welfare of Japan. The experimental protocol is shown in Supplementary Fig. 1.

### Bile and serum analyses

Daily bile flow from the biliary decompression catheter (ENBD or PTBD) was recorded for each patient. Bile samples were collected from the biliary decompression tube (ENBD or PTBD) before ICKT administration, 3 h after ICKT administration, and 24 h after ICKT administration (Supplementary Fig. 1). Blood samples were collected using a 21-G needle and vacuum collection tubes. After collection, blood samples were centrifuged at 3100 rpm at 24 °C for 10 min to prepare serum. The serum samples were then stored at − 70 °C until use. Biochemical analyses of the serum samples were performed using an automatic analyzer (LABOSPECT 008, Hitachi Ltd., Tokyo, Japan). Alanine aminotransferase (ALT), aspartate aminotransferase (AST), alkaline phosphatase (ALP) and γ-glutamyltransferase (γ-GTP) in serum were measured using commercially available assay kits (Shino-Test, Kanagawa, Japan). Total and direct bilirubin (T-Bil and D-Bil) were measured using commercially available assay kits (LSI Medience Corporation, Tokyo, Japan). The assays were performed according to the manufacturer’s instructions. Assays to measure bile acid concentrations, were performed by LSI Medience.

### Pharmacokinetic analysis of ICKT components

The plasma concentrations of all components were determined using LC–MS/MS. The instruments consisted of an Agilent 1290 UPLC system (Agilent Technologies, Santa Clara, CA, USA) with a 6500QTRAP triple quadrupole mass spectrometer fitted with a TurboIonSpray electrospray ionization interface (AB Sciex, Framingham, MA, USA). A CAPCELL CORE AQ column (150 × 2.1 mm i.d., 2.7 μm particle size; Shiseido, Tokyo, Japan) was used for all components. The mobile phase consisted of 10 mM ammonium acetate (solution A) and acetonitrile (solution B) at a flow rate of 0.3 mL/min.

For quantification of 6,7-dimethylesculetin, geniposide and genipin, 50 µL of plasma was mixed with 50 µL of water and acetonitrile (4:1, v/v), 10 µL of internal standard solution (100 ng/mL niflumic acid), and 300 µL of acetonitrile. The mixture was centrifuged at 1800×*g* at 4 °C for 15 min. The supernatant was collected in a test tube at 40 °C in a dry bath under a stream of nitrogen gas. The residue was resolved with 100 μL of water and acetonitrile (9:1, v/v). An aliquot (20 µL) of each sample was injected onto the analytical column.

#### Analysis of 6,7-dimethylesculetin and geniposide

The binary gradient elution was performed as follows: (1) start at 10% B from 0 to 2 min; (2) linear gradient from 10 to 35% B from 2 to 8 min; (3) maintained at 35% B from 8 to12 min. The mass spectrometer was operated in positive-ion mode. The high-purity nitrogen gas was composed of ion source gas 1, ion source gas 2, curtain gas, and collision-activated dissociation gas at pressures of 50, 70, and 30 psi respectively. The optimized TurboIonSpray voltage and temperature were set at 5500 V and 300 °C, respectively. The mass spectrometer was operated in the multiple reaction monitoring (MRM) mode, and the MRM-transitions were set at m/z 207.1–151.1 for 6,7-dimethylesculetin and m/z 406.1–226.9 for geniposide. The quantification ranges for 6,7-dimethylesculetin and geniposide were 0.0448–44.8 and 0.0416–41.6 ng/mL respectively.

#### Analysis of genipin

The binary gradient elution was performed as follows: (1) start at 10% B from 0 to 4 min; (2) linear gradient from 10% B to 35% B from 4 to 9 min; (3) maintained at 35% B from 9 to 13 min. The mass spectrometer was operated in negative-ion mode. The pressures of ion source gas 1, ion source gas 2, curtain gas and collision-activated dissociation gas were 70, 80, and 40 psi respectively. The optimized TurboIonSpray voltage and temperature were set at − 4500 V and 600 °C, respectively. MRM-transitions were set at m/z 225.0–122.9 for genipin. The quantification range for genipin was 0.107–42.8 ng/mL.

#### Analysis of geniposide and 6,7-dimethylesculetin

The binary gradient elution was performed as follows: (1) start at 10% B from 0 to 2 min; (2) linear gradient from 10 to 35% B from 2 to 8 min; and (3) maintenance at 35% B from 8 to 12 min. Mass spectrometry was performed in positive-ion mode. High-purity nitrogen gas was composed of ion source gas 1, ion source gas 2, curtain gas, and collision-activated dissociation gas at pressures of 50, 70, and 30 psi, respectively. The optimized TurboIonSpray voltage and temperature were set at 5500 V and 300 °C, respectively. The mass spectrometer was operated in multiple reaction monitoring (MRM) mode, and the MRM transition was set at m/z 406.1–226.9 for geniposide and m/z 207.1–151.1 for 6,7-dimethylesculetin. The quantification ranges for geniposide and 6,7-dimethylesculetin were 0.0416–41.6 and 0.0448–44.8 ng/mL respectively.

#### Analysis of genipin

The binary gradient elution was performed as follows: (1) start at 10% B from 0 to 4 min; (2) linear gradient from 10 to 35% B from 4 to 9 min; and (3) maintenance at 35% B from 9 to 13 min. The mass spectrometer was operated in negative-ion mode. The pressures of ion source gas 1, ion source gas 2, curtain gas and collision-activated dissociation gas were 70, 80, and 40 psi, respectively. The optimized TurboIonSpray voltage and temperature were set at − 4500 V and 600 °C, respectively. The MRM transition was set at m/z 225.0–122.9 for genipin. The quantification range for genipin was 0.107–42.8 ng/mL.

### Serum metabolome analysis using GC–MS/MS

Serum metabolome analysis using GC–MS/MS was performed according to the method described by Ohbuchi et al. (Ohbuchi et al. 2015) with minor modifications. To extract low**-**molecular weight metabolites for GC/MS analysis, 50 μL of plasma were mixed with 260 μL of a solvent mixture (MeOH:H2O:CHCl3 = 2.5:1:1) containing 10 μL of 0.5 mg/mL 2-isopropylmalic acid (Sigma–Aldrich, SL, USA) dissolved in distilled water, then the solution was shaken at 1400 rpm for 30 min at 37 °C before being centrifuged at 19,000×*g* for 3 min at 4 °C. A 150 μL-aliquot of the resulting supernatant was transferred to a clean tube and 140 μL of distilled water was added. After mixing, the solution was centrifuged at 19,000×g for 3 min at 4 °C, and 180 μL of the supernatant was transferred to a clean tube and lyophilized using a freeze dryer. For oximation, 80 μL of 20 mg/mL methoxyamine hydrochloride (Sigma–Aldrich) dissolved in pyridine was mixed with a lyophilized sample before being sonicated for 20 min using a water bath sonicator. Then, samples were shaken at 1200 rpm for 90 min at 30 °C. Next, 40 μL of *N*-methyl-*N*-trimethylsilyl-trifluoroacetamide (MSTFA) (GL Science, Tokyo, Japan) was added for derivatization and the mixture was incubated at 1200 rpm for 30 min at 37 °C. The mixture was then centrifuged at 19,000×*g* for 3 min and the resulting supernatant was subjected to measurement by GC/MS.

GC/MS measurements were performed using a GCMS-TQ8040 (Shimadzu, Kyoto, Japan) with a fused silica capillary column (DB-5; inner diameter: 30 m × 0.25 μm, film thickness: 1 µm; Agilent Co.). Chromatogram acquisition, detection of mass spectral peaks, and their waveform processing were performed using Shimadzu GCMSsolution software. The identification of low**-**molecular weight metabolites was performed using the Smart Metabolites Database (Shimadzu), which contains a mass spectral library; method files that specify the above-described analytical conditions; and data analysis parameters for 475 compounds, such as amino acids, fatty acids, and organic acids. All detected peaks were checked and corrected using GCMSsolution software. The peak intensity of each quantified ion was calculated and normalized to that of 2-isopropylmalic acid, which was used as an internal standard. Further analysis was performed using normalized values.

### Serum metabolome analysis using LC–MS/MS

Serum metabolome analysis using LC–MS/MS was performed according to the method described by Yamada et al. (Yamada et al. 2015) with minor modifications. To extract low**-**molecular weight metabolites for LC/MS analysis, 1 mL of methanol with internal standard mixture was added to 200 μL of plasma sample. The mixture was mixed for 5 min at room temperature and centrifuged at 15,000×*g* for 3 min. The supernatant was diluted with 4 mL of 0.1% formic acid in water and gently mixed. Then, the mixture was then loaded onto a preconditioned solid-phase extraction cartridge (STRATA-X, 10 mg/1 mL, Phenomenex, Torrance, CA). The cartridge was washed with 1 mL each of 0.1% formic acid and, 15% ethanol. The lipids were eluted with 250 μL of 0.1% formic acid in methanol. The eluent was evaporated using a vacuum evaporator and reconstituted in 20 μL of methanol. A 5 μL aliquot of sample was injected for analysis.

The LC/MS system consisted of two LC-30AD pumps, an SIL-30AC auto-sampler, a CTO-20A column oven, a CBM-20A system controller, and a triple quadrupole mass spectrometer LCMS-8050 (Shimadzu, Kyoto, Japan). A reversed-phase column (Kinetex C8, 2.1 × 150 mm, 2.6 μm, Phenomenex, Torrance, CA) was used for chromatographic separation. Chromatogram acquisition, detection of mass spectral peaks, and their waveform processing were performed using Labsolutions LCMS software, Labsolutions Insight software, and LC/MS/MS Method Package for Lipid Mediators Ver.2 (Shimadzu). The method package contains a mass spectral library; method files that specify the analytical conditions; and the data analysis parameters for 158 lipid mediators derived from arachidonic acid, eicosapentaenoic acid, docosahexaenoic acid, and other compounds. All detected peaks were checked and corrected using Labsolutions Insight software. The peak area of each quantified ion was calculated and normalized to those of an internal standard mixture contained containing 0.5 ng/μL each of the tetranor-PGEM-d_6_, TXB2-d_4_, PGE2-d_4_, PGD2-d_4_, LTC4-d_5_, LTB4-d_4_, 5-HETE-d_8_ and 15-HETE-d_8_, 0.25 ng/μL of oleoylethanolamide (OEA)-d_4_, and 10 ng/μL AA-d_8_ in methanol. All internal standards were purchased from Cayman Chemical. Further analyses were performed using normalized values.

### Processing and statistical analysis

Processing of metabolomics data was performed using Microsoft Excel software. Missing values in the raw data were replaced by half of the minimum positive value for each metabolite, and these data were used for subsequent statistical analysis.

All data were expressed as mean ± SD or SE. The statistical significance of differences between two time points was evaluated by paired *t*-test using GraphPad Prism 6 (GraphPad Software, CA, USA). A probability of less than 0.05 was considered statistically significant. Principal component analysis (PCA) and volcano plot analysis were performed for the results of serum metabolome analyses. PCA and the calculation of Pearson correlation coefficients were performed with SIMCA 14 software (Umetrics, Sweden). Biomarker exploration was performed using the Biomarker Analysis module in MetaboAnalyst (http://www.metaboanalyst.ca/) (Xia et al. 2012). The construction of a correlation network was performed using Cytoscape software (http://www.cytoscape.org/). The generation of the receiver operating characteristics (ROC) curves was performed using GraphPad Prism 6.

## Results

### Patient characteristics

Samples from 37 patients were analyzed. The characteristics of the study patients are described in Table 1. The median age was 67 years. The disease diagnoses consisted of perihilar cholangiocarcinoma (86%) and other biliary tract carcinoma (10%). In most patients (95%), biliary drainage was performed by ENBD.

**Table 1:** Patient clinical characteristics (n = 37)

Age (y)	67 (64–74)
Gender (Male/female)	26/11
Diagnosis, n (%)	
Perihilar cholangiocarcinoma	32 (86%)
Distal cholangiocarcinoma	2 (5%)
Gallbladder carcinoma	2 (5%)
Hepatocellular carcinoma	1 (3%)
Method of bililary drainage, n (%)	
ENBD	35 (95%)
PTBD	2 (5%)
ICGR15 (%)	8.3 (6.0–11.2)
ICGK	0.166 (0.149–0.187)
Child Pugh score	
A	22 (59%)
B	14 (38%)
C	1 (3%)
Hepatitis B	1 (3%)
Hepatitis C	0

Continuous data are presented as median (interquartile range)

*ENBD* endoscopic nasal biliary drainage, *PTBD* percutaneous transhepatic biliary drainage, *ICGR15* indocyanine green retention rate at 15 min, *ICGK* plasma disappearance rate of indocyanine green

### Hepatobiliary marker trends before and after ICKT treatment

Administration of ICKT (2.5 g/packet, 3 packets/day) significantly increased bile flow and decreased serum ALT levels after 24 h (Table 2). Increased bile flow after ICKT treatment was observed in 73% of patients (27/37). Among patients with ALT levels above normal (> 30 IU/L), 40% (12/30) of patients showed a > 10% decrease in ALT. Other biliary markers did not show any change after ICKT administration.

**Table 2:** Hepatobiliary markers in bile and serum before and after treatment

	Before ICKT treatment	24 h after ICKT treatment	*P* value
Bile			
Total bilirubin (mg/dL)	46.5 ± 30.4	46.8 ± 27.7	0.903
Direct bilirubin (mg/dL)	43.2 ± 28.8	42.7 ± 26.3	0.848
Bile acid (µmol/dL)	9.30 ± 7.70	11.7 ± 12.0	0.178
**Bile flow per 24 h (mL)**	**480** ± **272**	**554** ± **278**	**0.005**
Serum			
AST (IU/L)	75 ± 84	61 ± 49	0.096
**ALT (IU**/**L)**	**99** ± **97**	**92** ± **87**	**0.023**
T-Bil (mg/dL)	3.9 ± 5.5	3.8 ± 5.7	0.253
D-Bil (mg/dL)	2.3 ± 4.3	2.3 ± 4.4	0.408
ALP (IU/L)	797 ± 522	798 ± 518	0.962
γ-GTP (IU/L)	339 ± 261	344 ± 275	0.696

Continuous data are presented as mean ± standard deviation

*P* values were calculated by paired *t*-test.Value listed in bold represent statistical significance (*P* < 0.05)

*AST* aspartate transferase, *ALT* alanine aminotransferase, *T-Bil* total bilirubin, *D-Bil* direct bilirubin, *ALP* alkaline phosphatase, *γ-GTP* γ-glutamyltransferase

### Serum concentrations of ICKT components

**Fig. 1:**
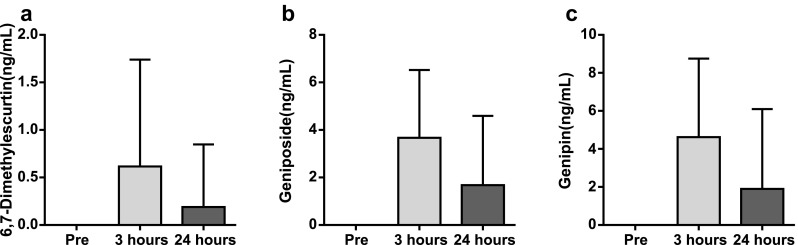
Serum concentrations of ICKT components **a** 6,7-dimethylesculetin, **b** geniposide and **c** genipin. The vertical bars represent mean ± SD (N = 37)

The serum concentrations of ICKT components such as 6,7-dimethylesculetin, geniposide, and genipin increased at 3 and 24 h after ICKT administration (Fig. 1). These results indicate that the major active constituents of ICKT were stably absorbed from the intestine. However, the serum levels of each constituent were widely variable among the patients studied. At 3 h after ICKT administration, the serum concentration of 6,7-dimethylesculetin ranged from below quantifiable limit (BQL) to 5.53 ng/ml; that of geniposide ranged from 0.198 to 13.0 ng/ml; and that of genipin ranged from 0.396 to 16.0 ng/ml. Serum concentrations of each constituent were not significantly correlated with changes in bile flow or ALT levels 24 h after ICKT administration (all correlation coefficients were below 0.2).

### Serum metabolome analysis

**Fig. 2:**
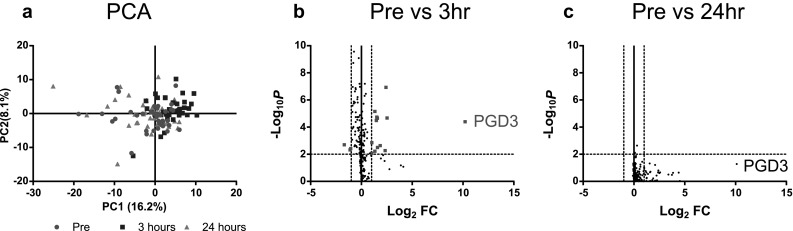
PCA score plot and volcano plots of the serum metabolome analysis. Serum metabolites compared before (designated as “Pre”) and 3/24 h after ICKT treatment are represented by PCA score plot (**a**) and volcano plots (**b, c**). **a** PCA score plot showed that the serum metabolites 3 h after ICKT administration differed from those of other groups. **b, c** Volcano plots mapped by the log_2_ fold change value for Pre/3 h (**c**), Pre/24 h and **c** versus-log_10_ *P* value of paired *t*-test. Red squares represent metabolites that met the criteria (*P* value < 0.01, fold change > 2 or < 0.5). A total of 16 metabolites met the criteria at 3 h post-ICKT administration, whereas no metabolites met the criteria at 24 h post-administration. Among them, the amount of PGD3 was drastically increased by the administration of ICKT. However, the peak was not derived from authentic PGD3

We next performed targeted metabolome analysis of common serum metabolites and lipid mediators. Low**-**molecular weight metabolites were extracted from serum, and common metabolites and lipid mediators in the serum were measured using GC–MS/MS and LC–MS/MS, respectively. A total of 217 metabolites (159 in GC–MS/MS analysis and 58 in LC–MS/MS analysis) were detected in serum before and after ICKT treatment (Supplementary Table 1). The PCA using the 217 metabolites as variables indicated that the pattern of serum metabolites at 3 h after ICKT administration differed from that before ICKT administration and 24 h after ICKT administration (Fig. 2a). The volcano plot analysis, which arranged the metabolites along axes of biological and statistical significance, indicated that there were 16 significantly changed metabolites, which fulfilled the criteria (fold change > 2.0 or < 0.5, *P* < 0.01), at 3 h after ICKT administration (Fig. 2b). Among them, the concentrations of 13 metabolites were significantly increased, whereas those of 3 metabolites were significantly decreased (Supplementary Table 2). These changes mostly subsided after 24 h (Fig. 2c). It should be noted that all patients had breakfast immediately after the administration of ICKT. Therefore, we could not determine whether most of the changes in serum metabolites from pretreatment to 3 h post-administration were caused by ICKT administration or meal ingestion. Among those changes, the serum level of prostaglandin D3 (PGD3) was drastically increased by the administration of ICKT and maintained at a high level even after 24 h (Fig. 2b, c). However, confirmation analysis using standard PGD3 demonstrated that the peak that was expected to be induced by ICKT administration did not coincide with that of the PGD3 standard (Supplementary Fig. 2), indicating that the metabolite was not authentic PGD3, even though the metabolite had the same mass transition as PGD3.

### Identification and evaluation of candidate biomarkers

**Fig. 3:**
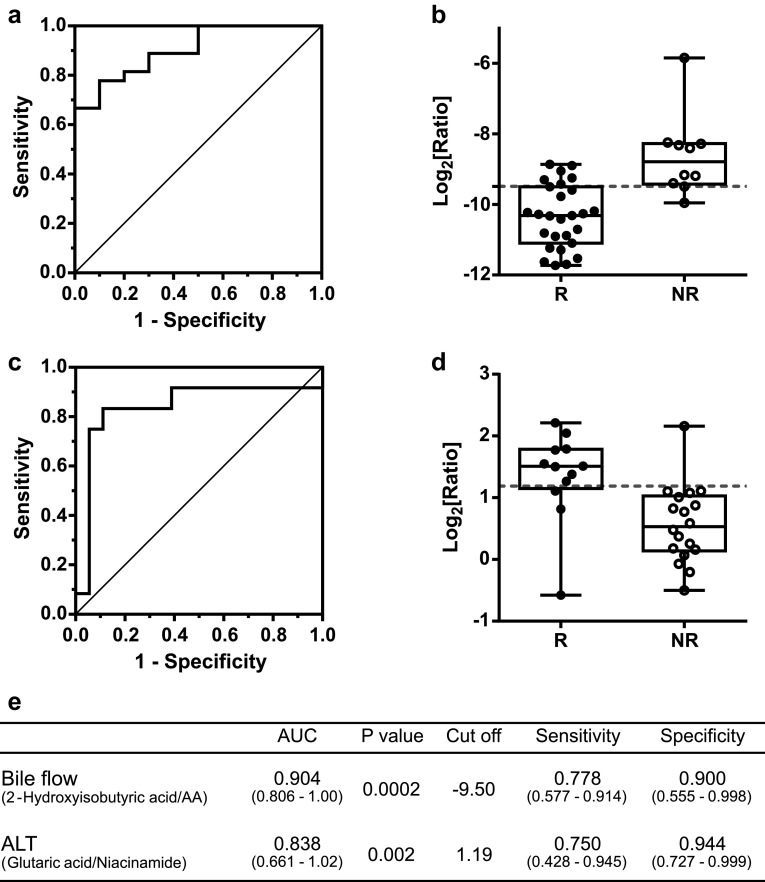
ROC analysis of biomarker candidates for ICKT efficacy on bile flow and serum ALT. ROC curves and box plots of biomarker candidates which classify poor and good responders for the improvement of bile flow (**a, b**) and serum ALT level (**c, d**) are represented. Each of selected cutoff values was represented by a red dotted line in (**b, d**). R and NR represent responder group and non-responder group, respectively. Performance indices [area under the ROC curve (AUC), *P* value, cut**-**off value, sensitivity and specificity for each biomarker candidates] are summarized in (**e**). The numbers in parentheses represent the 95% confidence interval

Candidate biomarkers for prediction of ICKT efficacy were explored using the Biomarker Analysis module in MetaboAnalyst. When ICKT responders were defined according to an increase in bile volume, the ratio of 2-hydroxyisobutyric acid to arachidonic acid displayed good performance indices (area under curve [AUC] of ROC curve was > 0.90, *P* < 0.001) (Fig. 3a, b). When ICKT responders were defined according to a decrease in ALT of > 10% among 30 patients with abnormal ALT (> 30 IU/L), the ratio of glutaric acid to niacinamide also exhibited good performance (AUC of ROC curve was > 0.85, *P* < 0.01) (Fig. 3c, d). The sensitivity and specificity of these biomarker candidates are summarized in Fig. 3e.

### Correlation analysis

**Fig. 4:**
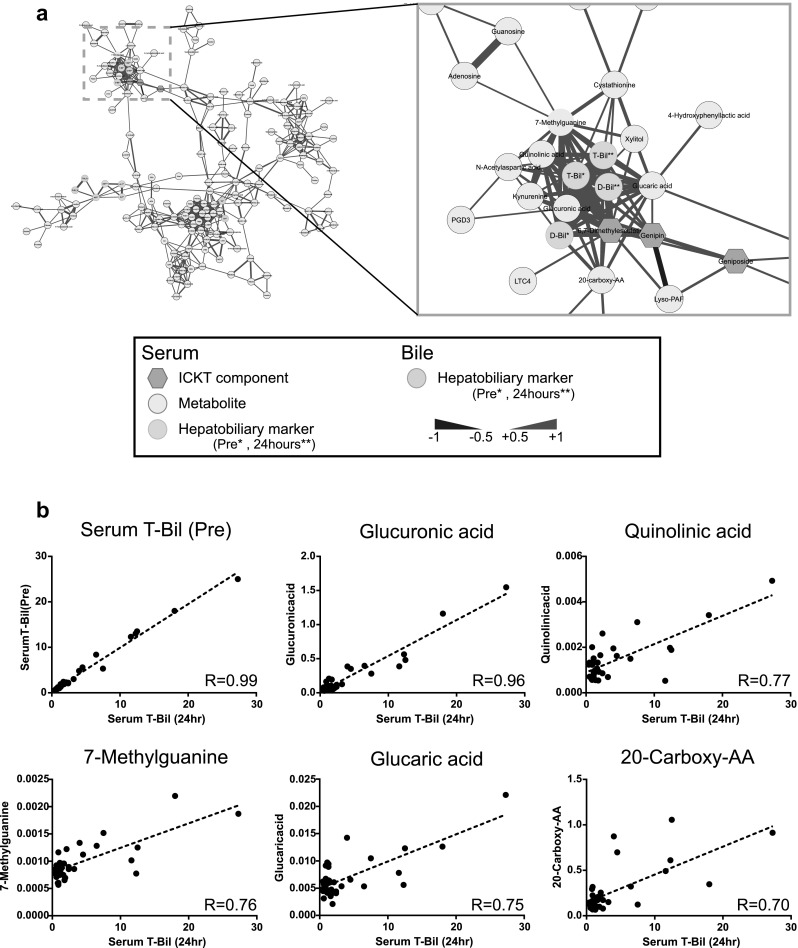
Correlation analysis of serum and biliary hepatobiliary biomarkers (Pre and 24 h), serum ICKT components (3 h) and serum metabolites (Pre). **a** A correlation network was constructed using the Pearson correlation coefficient (R > 0.6 or < − 0.6). Red and blue colors at edge represent positive and negative correlation, respectively. The width of the edge represents the strength of correlation. The whole correlation network is based on hepatobiliary markers, serum concentration of ICKT components 3 h after ICKT treatment, and serum metabolites before ICKT treatment (designated as “Pre”). The cluster consisting of ICKT components is highlighted by an orange dotted line (left panel) and enlarged (right panel). **b** Scatter plots between serum T-Bil 24 h after administration and serum T-Bil and metabolites before administration. Correlation coefficients are presented in the graph

To obtain information about the pathogenesis of jaundice with respect to metabolomics, Pearson correlation analyses of various clinical metrics including patient characteristics, biochemical, and hepatobiliary markers (the list of analyzed parameters is shown in Supplementary Table 3), metabolites (Pre), and ICKT components (3 h) were performed. The analyses enabled the extraction of clusters with close interrelationships among the parameters, which generally contain novel unknown relationships among apparently unconnected metabolites/markers as well as known or easily acceptable relationships among well-connected metabolites/markers. When the correlation network was constructed using a Pearson correlation coefficient greater than 0.6 (R > 0.6 or R < − 0.6), several clusters of correlations were identified (Fig. 4a). In one such prominent cluster, glucuronic acid, quinolinic acid, 7-methylguanine, glucaric acid, and 20-carboxy arachidonic acid measured at the beginning of the observation period were found to be closely correlated with serum total bilirubin prior to treatment and after 24 h post-treatment (Fig. 4b). In particular, glucuronic acid showed an extremely high correlation (R = 0.96) with total bilirubin. We confirmed that there were no outliers in the parameters for each factor by scatter plot analysis (Fig. 4b).

## Discussion

Inchinkoto (ICKT) is one of the herbal medicines that are commonly used as a hepatoprotective agent in Japan. The biological properties of ICKT and its major active constituents, 6,7-dimethylesculetin and genipin, have been studied in numerous experiments involving cultured cells and laboratory animals (Amamoto et al. 1996; Yamamoto et al. 2000; Leu et al. 2003; Huang et al. 2004; Shoda et al. 2004; Okada et al. 2007). Moreover, there are several clinical reports showing a clinical benefit of ICKT as a hepatoprotective agent (Kobayashi et al. 2001; Kaiho et al. 2008; Watanabe et al. 2009). These reports indicated anti-cholestatic (Shoda et al. 2004; Okada et al. 2007), anti-apoptotic (Amamoto et al. 1996; Yamamoto et al. 2000; Takeuchi et al. 2005), anti-inflammatory (Yamashiki et al. 2000; Arab et al. 2009; Kawai et al. 2010), and anti-fibrotic effects (Sakaida et al. 2003; Imanishi et al. 2004; Inao et al. 2004) of ICKT and/or its major component genipin. Although these data support the clinical efficacy of ICKT in patients with various liver diseases, especially for those with biliary obstruction, there is considerable variation in the therapeutic response to ICKT. It has been reported that the concentrations of biliary T-Bil and bile acid are increased in about 70% of patients with biliary drainage (due to biliary obstruction by malignancies) 72 h after oral administration of ICKT (Watanabe et al. 2009). However, in the remaining 30% of patients, ICKT did not exert a choleretic effect. In the present study, the concentrations of biliary T-Bil and bile acid were not significantly affected. However, the treatment period (24 h) was shorter than in the previous study (72 h). Further, it should be noted that the net amount of T-Bil and bile acid excreted into the bile would be higher because the bile volume after 24 h increased significantly. Thus the choleretic effect of ICKT has essentially been validated again in this study and, importantly, a similar response rate was obtained (the bile volume increased in 73% of patients). The mechanism underlying the variable response to ICKT was unknown. Therefore, in this study, we sought to identify a biomarker that could differentiate between ICKT responders and non-responders. Furthermore, a comprehensive correlation analysis was performed in order to obtain insights regarding for the pathogenic mechanism of jaundice.

The pharmacokinetic analysis was performed using samples obtained prior to treatment and at 3 and 24 h after treatment. We then measured the serum concentration of genipin, which would be produced from geniposide by intestinal microbiota. Glychyrrhetinic acid, which is an important active substance of licorice root, is also produced from glychyrrhizin in the same manner as genipin. A clinical study reported that glychyrrhetinic acid can be detected 3 h or more after administration of licorice root containing the Kampo medicine, rikkunshito (Kitagawa et al. 2015). Additionally, we have evaluated some herbal medicines in mice and rats using plasma metabolome analysis (Ohbuchi et al. 2015; Nishi et al. 2017). In these studies, a number of metabolites significantly changed at 2–4 h after administration. Therefore, we decided to obtain samples 3 h after administration for pharmacokinetic and metabolome analyses. Sampling at 24 h after administration was done to evaluate the effects of repeated ICKT administration. In the pharmacokinetic analysis, genipin as well as geniposide, and 6,7-dimethylesculetin could be detected in the serum. However, none of them showed an association with the decrease in ALT level and increase in bile flow, suggesting that the concentrations of active compounds in peripheral blood could not predict ICKT efficacy. Such results are understandable because previous animal studies suggested that ICKT compounds reach hepatocytes primarily via the portal vein after absorption from the intestines, and the discharge to the peripheral blood without conjugation should be modest. It is possible that the effect of ICKT is be correlated with pharmacokinetic parameters of ICKT components such as AUC and C_max_.

In the metabolome analysis, we identified 16 metabolites that significantly changed at 3 h after ICKT treatment based on the volcano plots (Fig. 2b). Unfortunately, we could not determine whether these metabolites were influenced by ICKT administration or meal intake because all patients had eaten breakfast immediately after ICKT administration. Among the 16 metabolites, a PGD3-like molecule showed the largest peak with more than a 1000-fold change. It was shown that the PGD3-like molecule had the same mass transition as that of PGD3. The increased serum concentration of the PGD3-like molecule was sustained even at 24 h post-treatment, at which time (just before breakfast), the amounts of most of the other metabolites that were increased at 3 h had subsided. Additionally, in a rat study, the PGD3-like metabolite was also induced by ICKT administration but not by vehicle administration (Supplementary Fig. 2). Therefore, it can be assumed that the metabolite was induced by ICKT administration rather than by meal ingestion. Although it is unknown whether the increase of the PGD3-like molecule was directly derived from the components of ICKT or ICKT induced a release of an endogenous PGD3-like molecule, the serum concentration of this PGD3-like molecule appears to be a promising compliance marker for ICKT treatment. It will be necessary to determine the structure and evaluate the biological activity of this metabolite in future investigations.

In the present study, biomarkers were explored using the Biomarker Analysis module in MetaboAnalyst. ROC analysis was then performed to address the possibility that the ratios of 2-hydroxyisobutyric acid to arachidonic acid and of glutaric acid to niacinamide might be candidate biomarkers for predicting ICKT efficacy on bile flow and ALT, respectively. Some of these candidate metabolites have been proposed as potential biomarkers for liver disease. For example, it has been suggested that 2-hydroxyisobutyric acid could be useful for the diagnosis of clinical non-alcoholic fatty liver disease (Miccheli et al. 2015) and diabetes mellitus (Li et al. 2009). Glutaric acid has also been identified as a potential biomarker for dimethylnitrosamin-induced hepatic fibrosis (Ju et al. 2013). Arachidonic acid is a key inflammatory intermediate. Niacinamide might play a role in the development of diabetes (Knip et al. 2000) and liver damage (Winter and Boyer 1973). Although these metabolites would be involved in liver disease and inflammation, the mechanisms by which they would relate to the efficacy of ICKT is largely unclear. Therefore, further investigations including the validation of these biomarkers are required.

Pearson correlation analysis revealed that metabolites such as glucuronic acid, quinolinic acid, 7-methylguanine, glucaric acid and 20-carboxy arachidonic acid are closely correlated with serum total bilirubin after 24 h (Fig. 4). Among them, glucuronic acid showed a very high correlation with serum total bilirubin (R = 0.96) suggesting that the metabolite may be profoundly involved in the pathogenic mechanisms of bilirubin overflow to the peripheral blood.

Glucaric acid is a metabolite of glucuronic acid and, therefore, the cluster may represent, at least in part, glucuronic acid metabolism. Glucuronic acid is involved in the process of glucuronidation, which is a major function of the liver and contributes to the elimination of various substances from the body through the bile. Impaired glucuronidation is observed in patients with advanced cirrhosis (Verbeeck 2008) and other types of hepatic dysfunction (Morgan and McLean 1995; Elbekai et al. 2004). The increased serum glucuronic acid concentration, therefore, may have been induced by some defect in the hepatic glucuronidation system. In accordance with this presumption, we detected unconjugated genipin in sera in the present study. Although no human pharmacokinetic studies of genipin have been published previously, animal studies suggest that orally administered geniposide, as a single compound or in the form of a crude herbal medicine, is converted to genipin in the gut, transported to the liver via the portal vein, and mostly conjugated with glucuronic acid; the majority of genipin detected in rat sera was is in the conjugated form (Han et al. 2011; Ding et al. 2013). By contrast, in the present study, a rather small amount of conjugated genipin was found in the peripheral blood, and in 11 patients, conjugated genipin was virtually undetectable (data not shown). These results suggest that obstructive jaundice may affect glucuronidation of genipin via altering glucuronic acid metabolism, and consequently, the detoxification of xenobiotic substances.

Additionally, urinary glucaric acid is a known biomarker for cytochrome P450 activation. Serum levels of quinolinic acid have been shown to be correlated with the severity of hepatic dysfunction in patients with liver cirrhosis (Lahdou et al. 2013). Collectively, the correlation cluster including serum bilirubin, glucuronic acid, glucaric acid and quinolinic acid appears to represent dysregulation of hepatic detoxification systems, particularly glucuronide metabolism/glucuronidation; however, the precise nature of the dysregulation and the role of other metabolites in the cluster remain to be determined. Furthermore, it should be noted that the cluster did not correlate with other biochemical markers of liver failure; for example, the retention of indocyanine green at 15 min (the correlation coefficient between ICGR15 and glucuronic acid was 0.057). This suggests that the metabolites in the cluster can be developed as novel biomarkers for liver failure.

Finally, there was a limitation in this study. We could not evaluate the effect of ICKT on serum metabolites because all patients had eaten breakfast immediately after ICKT administration. This aspect of the experimental design would need to be improved in order to clarify the effect of ICKT administration. For example, ICKT administration and blood sampling could be performed during a fasting period. It would also be advisable to track serum metabolomics and pharmacokinetics for a longer duration, since the effect of ICKT would become clearer with a longer dosing period. We plan to perform a future validation study in which the points raised above will be addressed.

## Conclusions

In conclusion, ICKT treatment in obstructive jaundice patients with biliary drainage reduced serum ALT levels and increased bile flow after 24 h. The present study provides biomarker candidates for ICKT responders, although further extensive validation studies are warranted. Additionally, glucuronic acid and presumably dysregulation of glucuronide-related detoxification systems have been found to be deeply involved in the pathogenesis of jaundice. The present study may open the way to the development of more efficient, “tailor-made” therapy with ICKT.

## Electronic supplementary material

Below is the link to the electronic supplementary material.

Supplementary material 1 (DOCX 214 KB)

Supplementary material 2 (DOCX 40 KB)
