# Correction to: Exploration of serum biomarkers for predicting the response to Inchinkoto (ICKT), a Japanese traditional herbal medicine

**DOI:** 10.1007/s11306-018-1407-z

**Published:** 2018-09-11

**Authors:** Masahito Uji, Yukihiro Yokoyama, Katsuya Ohbuchi, Kazuaki Tsuchiya, Chiharu Sadakane, Chika Shimobori, Masahiro Yamamoto, Masato Nagino

**Affiliations:** 1 Division of Surgical Oncology, Department of Surgery, Nagoya University Graduate School of Medicine, 65 Tsurumai-cho, Showa-ku, Nagoya, Japan; 2 Tsumura Research Laboratories, Tsumura & Co., Ami, Japan

## Correction to: Metabolomics (2017) 13:155 10.1007/s11306-017-1292-x

The article Exploration of serum biomarkers for predicting the response to Inchinkoto (ICKT), a Japanese traditional herbal medicine, written by Masahito Uji, Yukihiro Yokoyama, Katsuya Ohbuchi, Kazuaki Tsuchiya, Chiharu Sadakane, Chika Shimobori, Masahiro Yamamoto, Masato Nagino, was originally published electronically on the publisher’s internet portal (currently SpringerLink) on 8 November 2017 without open access. With the author(s)’ decision to opt for Open Choice the copyright of the article changed on 24 July 2018 to © The Author(s) [2018] and the article is forthwith distributed under the terms of the Creative Commons Attribution 4.0 International License (http://creativecommons.org/licenses/by/4.0/), which permits use, duplication, adaptation, distribution and reproduction in any medium or format, as long as you give appropriate credit to the original author(s) and the source, provide a link to the Creative Commons license and indicate if changes were made.

The original article has been corrected.

