# Supplementary material for: Exploration of serum biomarkers for predicting the response to Inchinkoto (ICKT), a Japanese traditional herbal medicine
Source: Metabolomics. 2017 Nov 8;13(12):155. doi: 10.1007/s11306-017-1292-x (PMC6153689; doi:10.1007/s11306-017-1292-x)
Supplement: Supplementary file 1 — Supplementary material 1 (DOCX 214 KB) [file 11306_2017_1292_MOESM1_ESM.docx]

**Exploration of serum biomarkers for predicting the response to Inchinkoto (ICKT), a Japanese traditional herbal medicine**

Masahito Uji, MD^1^, Yukihiro Yokoyama, MD^1^, Katsuya Ohbuchi, PhD^2^, Kazuaki Tsuchiya, MS^2^, Chiharu Sadakane, PhD^2^, Chika Shimobori, MS^2^, Masahiro Yamamoto, PhD^2^, Masato Nagino, MD^1^

1. Division of Surgical Oncology, Department of Surgery, Nagoya University Graduate School of Medicine, Nagoya, Japan.
2. Tsumura Research Laboratories, Tsumura & Co., Ami, Japan

**Address correspondence to:**

Yukihiro Yokoyama, M.D., Ph.D.

Division of Surgical Oncology, Department of Surgery

Nagoya University Graduate School of Medicine

65 Tsurumai-cho, Showa-ku, Nagoya, 466-8550, Japan

E-mail address: [yyoko@med.nagoya-u.ac.jp](mailto:yyoko@med.nagoya-u.ac.jp)

Tel: +81 52-744-2222, Fax: +81 52-744-2230


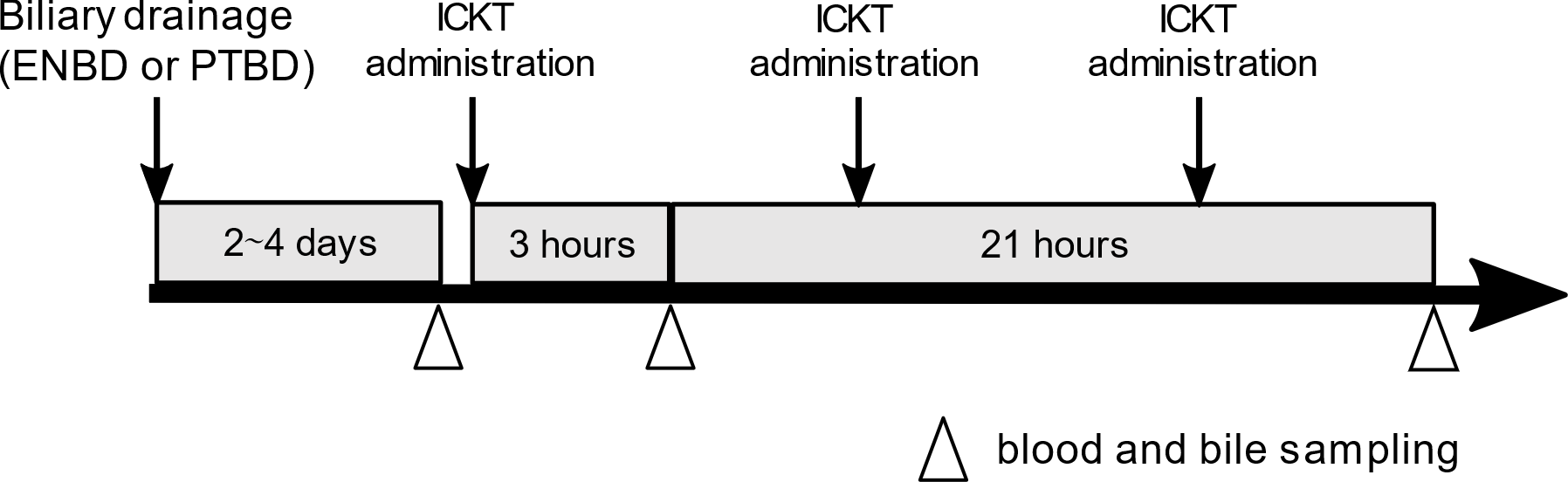


**Supplementary Fig. 1**

**Sampling schedule.** Biliary drainage was performed 2 to 4 days before blood and bile sampling. The blood and bile samples were collected before and 3 hours/24 hours after ICKT administration.

**Supplementary Fig. 2 Confirmation analysis of PGD3 detected in metabolome analysis**

The metabolite having PGD_3_-specific mass transition (349.2 > 269.2, black chromatogram) was detected in the serum from the patient 3 hours after ICKT administration (“Human 3 hours after ICKT admin”) but not from serum before ICKT treatment (“Human Pre”). However, the peak was not detected by another PGD_3_ specific mass transition (349.2 > 233.2, purple chromatogram). This phenomenon was also observed in a rat study. SD rats were orally administered vehicle (“Rat Control”) or 2 g/kg ICKT extract (“Rat ICKT”), and blood samples were then collected 3 hours after administration. Additionally, PGD_3_ standard was added to ~~the~~ plasma from the rat treated with ICKT. The chromatogram of PGD_3_-spiked plasma is shown in “Rat ICKT plasma with PGD_3_ std”. The peak caused by ICKT administration and that of PGD_3_ standard are indicated by red and blue arrowheads, respectively. Since they have different retention times, the metabolite arising from ICKT administration does not appear to be PGD_3_.
