# Supplementary material for: Exploration of serum biomarkers for predicting the response to Inchinkoto (ICKT), a Japanese traditional herbal medicine
Source: Metabolomics. 2017 Nov 8;13(12):155. doi: 10.1007/s11306-017-1292-x (PMC6153689; doi:10.1007/s11306-017-1292-x)
Supplement: Supplementary file 2 — Supplementary material 2 (DOCX 40 KB) [file 11306_2017_1292_MOESM2_ESM.docx]

**Exploration of serum biomarkers for predicting the response to Inchinkoto (ICKT), a Japanese traditional herbal medicine**

Masahito Uji, MD^1^, Yukihiro Yokoyama, MD^1^, Katsuya Ohbuchi, PhD^2^, Kazuaki Tsuchiya, MS^2^, Chiharu Sadakane, PhD^2^, Chika Shimobori, MS^2^, Masahiro Yamamoto, PhD^2^, Masato Nagino, MD^1^

1. Division of Surgical Oncology, Department of Surgery, Nagoya University Graduate School of Medicine, Nagoya, Japan.
2. Tsumura Research Laboratories, Tsumura & Co., Ami, Japan

**Address correspondence to:**

Yukihiro Yokoyama, M.D., Ph.D.

Division of Surgical Oncology, Department of Surgery

Nagoya University Graduate School of Medicine

65 Tsurumai-cho, Showa-ku, Nagoya, 466-8550, Japan

E-mail address: [yyoko@med.nagoya-u.ac.jp](mailto:yyoko@med.nagoya-u.ac.jp)

Tel: +81 52-744-2222, Fax: +81 52-744-2230

Supplementary Table 1. Changes of metabolites detected in the serum Pre and 3 hours after ICKT treatment.

|  | 3 hours / Pre | | | 24 hours / Pre | | |
| --- | --- | --- | --- | --- | --- | --- |
|  | Fold change | -Log_10_*P* | Fold change | | -Log_10_*P* |  |
| Pyruvic acid  Lactic acid  2-Hydroxyisobutyric acid  Glycolic acid  Caproic acid  2-Ketobutyric acid  Alanine  2-Keto-isovaleric acid  Hydroxylamine  2-Hydroxybutyric acid  3-Hydroxypropionic acid  3-Hydroxybutyric acid  2-Hydroxyisovaleric acid  2-Aminobutyric acid  3-Methyl-2-oxovaleric acid  3-Hydroxyisovaleric acid  2-Ketoisocaproic acid  Valine  Urea  Benzoic acid  Octanoic acid  Glycerol  2-Aminoethanol  Leucine  Phosphoric acid  Acetylglycine  Isoleucine  Nicotinic acid  Proline  Phenylacetic acid  Succinic acid | 2.77  0.98  0.97  1.00  0.98  2.03  1.08  0.98  0.94  0.81  1.02  0.54  0.98  0.93  0.89  1.00  0.89  0.93  1.01  0.96  1.16  0.92  0.88  0.84  0.98  0.81  0.88  1.29  1.16  1.04  0.93 | 4.49  0.91  0.78  0.15  0.69  2.85  1.93  0.65  2.76  3.91  0.25  3.89  0.75  2.88  2.43  0.35  2.71  2.88  0.13  1.93  1.71  1.33  3.99  4.75  0.59  3.90  2.46  1.63  5.72  0.08  1.55 | 1.45  1.09  0.99  1.02  1.02  1.20  1.03  1.04  1.02  0.95  1.03  1.49  1.00  0.96  1.02  1.04  0.99  1.01  1.01  1.01  1.04  1.26  1.03  1.02  1.04  0.98  1.04  1.56  1.03  1.05  1.01 | | 0.58  0.35  0.91  0.43  0.23  0.07  0.08  0.02  0.38  0.90  0.12  0.30  0.18  1.22  0.07  0.04  0.55  0.08  0.14  0.22  0.36  0.48  0.01  0.13  0.43  0.45  0.45  1.08  0.60  0.90  0.23 |  |

Supplementary Table 1 (continued)

|  | 3 hours / Pre | | | 24 hours / Pre | | |
| --- | --- | --- | --- | --- | --- | --- |
|  | Fold change | -Log_10_*P* | Fold change | | -Log_10_*P* |  |
| Glycine  Methylsuccinic acid  Glyceric acid  Fumaric acid  Uracil  Nonanoic acid  Serine  Threonine  Glutaric acid  3-Aminopropanoic acid  Decanoic acid  3-Aminoisobutyric acid  Citramalic acid  Malic acid  Niacinamide  Adipic acid  Threitol  Dihydrouracil  meso-Erythritol  N-Acetylserine  3-Aminoglutaric acid  Aspartic acid  Methionine  4-Hydroxyproline  5-Oxoproline  Cytosine  4-Aminobutyric acid  Pyrogallol  Threonic acid  Cysteine  2-Hydroxyglutaric acid  Creatinine | 0.88  1.03  1.03  1.06  1.02  0.94  0.83  0.90  0.97  1.19  1.18  0.88  1.23  1.02  0.75  1.01  1.21  0.94  1.28  1.07  0.60  0.60  0.91  0.90  0.88  2.99  1.05  1.30  1.08  1.01  0.93  1.04 | 7.19  0.12  0.40  0.45  0.33  1.18  9.08  6.97  0.79  0.74  0.93  3.15  3.49  0.31  4.55  0.73  3.60  2.12  0.04  1.48  5.88  5.87  3.40  4.88  4.50  2.49  0.09  0.40  4.18  0.18  2.12  0.01 | 1.02  1.05  1.08  1.10  1.23  1.01  1.02  1.01  0.99  1.01  1.06  1.03  1.07  1.04  1.11  1.04  1.10  1.05  1.07  1.05  1.16  1.16  1.01  1.04  1.06  2.92  1.13  1.95  1.05  1.09  1.03  0.98 | | 0.34  0.11  1.11  0.67  2.64  0.29  0.07  0.25  0.40  0.38  0.25  0.12  0.10  0.07  0.00  0.01  0.36  0.32  0.61  0.97  0.22  0.22  0.22  0.65  1.06  1.32  1.77  0.55  0.83  1.78  0.02  0.85 |  |

Supplementary Table 1 (continued)

|  | 3 hours / Pre | | | 24 hours / Pre | | |
| --- | --- | --- | --- | --- | --- | --- |
|  | Fold change | -Log_10_*P* | Fold change | | -Log_10_*P* |  |
| 2-Ketoglutaric acid  3-Phenyllactic acid  Hypotaurine  Cadaverine  Glutamic acid  4-Hydroxybenzoic acid  5-Aminovaleric acid  Phenylalanine  4-Hydroxyphenylacetic acid  Lauric acid  Xylose  Arabinose  Asparagine  N-Acetylaspartic acid  Ribulose  Ribose  Xylulose  Taurine  Xylitol  1,6-Anhydroglucose  Arabitol  Ribitol  Aconitic acid  2-Deoxy-glucose  Quinolinic acid  Fucose  Putrescine  Dihydroxyacetone phosphate  Glycerol 3-phosphate  Glutamine  Azelaic acid  Dihydroorotic acid | 1.05  1.19  0.76  1.06  0.67  0.78  1.05  0.93  1.14  0.90  2.03  1.27  0.92  1.04  1.04  0.62  0.93  0.77  1.14  1.77  1.17  1.16  1.36  0.99  1.16  1.03  0.89  0.97  0.97  0.94  1.12  1.06 | 0.04  1.74  4.75  0.37  6.88  5.81  0.04  3.01  0.43  1.46  2.07  7.19  2.94  0.15  0.08  2.59  1.89  3.96  3.79  2.06  0.80  0.82  6.45  0.54  2.31  0.78  2.34  1.00  0.97  2.87  0.23  0.19 | 1.09  1.19  1.11  1.05  1.13  1.08  1.11  1.02  1.28  1.06  1.17  1.08  1.03  1.04  1.16  1.70  1.14  1.15  1.06  1.45  1.10  1.08  1.08  1.01  1.08  1.05  1.03  1.09  1.10  0.97  1.15  1.09 | | 0.35  0.98  0.27  0.16  0.66  0.31  0.39  0.13  0.01  0.15  0.02  1.36  0.10  0.11  1.87  0.07  1.30  0.18  0.55  0.12  0.06  0.05  1.07  0.25  0.83  2.07  0.23  0.53  0.68  1.16  0.06  1.10 |  |

Supplementary Table 1 (continued)

|  | 3 hours / Pre | | | 24 hours / Pre | | |
| --- | --- | --- | --- | --- | --- | --- |
|  | Fold change | -Log_10_*P* | Fold change | | -Log_10_*P* |  |
| O-Phosphoethanolamine  2-Aminopimelic acid  Isocitric acid  Hypoxanthine  Citric acid  Arginine  Ornithine  Dopamine  Myristic acid  Hippuric acid  Pyridoxal  1,5-Anhydro-glucitol  Tagatose  Psicose  Vanilmandelic acid  Fructose  Mannose  4-Hydroxyphenyllactic acid  Lysine  Tyramine  Histidine  Glucosamine  Glucose  N6-Acetyllysine  1-Hexadecanol  Tyrosine  Mannitol  Glucuronic acid  Sorbitol  Epinephrine  Galactosamine  Indol-3-acetic acid | 1.23  1.03  1.12  0.68  1.03  0.97  0.94  0.94  0.84  1.23  1.07  1.00  1.06  2.96  1.26  5.78  0.94  1.01  0.95  0.95  1.01  1.02  0.99  1.06  1.00  1.00  3.59  1.08  1.65  0.92  1.19  1.34 | 0.34  0.11  2.25  4.38  0.11  1.14  1.77  1.61  3.29  3.32  1.79  0.25  5.15  4.66  4.24  4.67  1.15  0.01  2.07  2.20  0.01  0.29  0.24  4.66  0.11  0.21  2.64  0.78  0.22  0.65  2.64  3.84 | 1.56  1.00  1.02  1.29  1.00  1.03  1.05  1.06  1.08  0.99  1.03  1.01  1.01  1.22  1.06  1.27  0.96  1.01  1.02  1.03  1.02  1.00  1.01  1.01  1.09  1.01  4.22  1.03  1.88  0.93  1.04  1.13 | | 1.24  0.33  0.05  0.35  0.33  0.61  0.47  0.45  0.14  0.26  0.45  0.13  0.65  0.37  0.19  0.37  0.77  0.06  0.11  0.23  0.04  0.17  0.28  0.08  0.06  0.06  1.21  0.33  0.00  0.41  0.16  0.54 |  |

Supplementary Table 1 (continued)

|  | 3 hours / Pre | | | 24 hours / Pre | | |
| --- | --- | --- | --- | --- | --- | --- |
|  | Fold change | -Log_10_*P* | Fold change | | -Log_10_*P* |  |
| Pantothenic acid  Palmitoleic acid  Xanthine  Gluconic acid  Palmitic acid  Glucaric acid  Uric acid  Inositol  Margaric acid  Octadecanol  Norepinephrine  Kynurenine  Linoleic acid  Oleic acid  7-Methylguanine  Cystathionine  Stearic acid  Tryptophan  Cystine  2'-Deoxyuridine  Uridine  5-Methoxytryptamine  Inosine  Adenosine  Sucrose  Lactose  Guanosine  Trehalose  Maltose  Lactitol  8-iso-PGF3a  TXB2 | 0.97  0.57  0.68  1.10  0.93  1.10  1.06  1.24  0.92  0.99  0.91  1.14  0.65  0.58  1.09  1.21  0.97  1.13  1.06  0.76  0.77  1.23  1.33  14.8  4.40  5.35  5.07  1.57  1.63  1.47  1.18  3.85 | 0.50  3.96  6.79  2.80  2.19  1.93  2.18  6.31  1.99  0.39  2.04  3.38  3.45  4.02  1.45  1.59  0.91  2.73  3.78  6.05  6.21  3.40  1.52  1.18  1.93  6.93  2.27  0.34  0.25  0.18  0.74  1.65 | 1.06  1.36  1.01  1.02  1.12  1.03  1.05  1.09  1.11  1.12  1.16  0.99  1.17  1.23  1.07  1.00  1.11  1.06  1.06  1.05  1.05  1.11  2.34  3.01  3.08  1.77  1.54  1.39  1.56  1.30  1.14  21.7 | | 0.36  0.00  0.30  0.59  0.69  0.19  0.77  1.24  0.55  0.22  0.48  0.06  0.05  0.01  1.01  0.84  0.82  0.61  2.12  0.19  0.26  0.81  0.03  0.04  0.72  1.45  0.27  0.03  0.02  0.32  0.13  0.67 |  |

Supplementary Table 1 (continued)

|  | 3 hours / Pre | | | 24 hours / Pre | | |
| --- | --- | --- | --- | --- | --- | --- |
|  | Fold change | -Log_10_*P* | Fold change | | -Log_10_*P* |  |
| PGD3  8-iso-15-keto-PGF2a  PGF2a  PGE2  11-dehydro-TXB2  PGE1  LTC4  LTE4  17,18-DiHETE  LTB4  14,15-DiHETE  12,13-DiHOME  9,10-DiHOME  5,6-DiHETE  14,15-DHET  12-HHT  11,12-DHET  8,9-DHET  9-HOTrE  20-carboxy-AA  5,6-DHET  18-HEPE  20-HETE  18-HETE  12-HEPE  16-HETE  5-HEPE  Lyso-PAF  13-HODE  9-HODE  20-HDoHE  15-HETE | 1207  1.00  0.99  1.03  1.32  1.46  4.96  1.22  1.03  1.25  0.84  2.89  2.55  0.70  0.84  1.98  0.78  1.20  5.90  1.02  1.05  0.96  2.54  0.97  0.47  1.70  0.59  1.09  0.95  1.38  0.72  0.66 | 4.37  0.13  0.96  1.61  1.72  1.73  1.36  1.64  0.28  2.81  2.88  4.51  2.20  2.60  3.76  1.83  4.71  2.10  0.81  0.17  2.69  3.39  5.25  1.33  2.32  0.58  5.73  1.10  1.14  0.05  2.88  2.55 | 1144  1.06  1.46  6.08  10.8  13.7  1.88  2.42  1.12  4.80  1.05  1.42  1.36  1.07  1.02  14.8  1.01  1.74  3.18  1.06  1.26  2.06  4.14  1.29  5.43  2.18  1.32  1.16  1.24  1.26  1.49  2.39 | | 1.28  0.19  0.75  0.69  0.51  0.56  0.47  0.26  0.25  0.48  0.07  0.08  0.19  0.10  0.11  0.67  0.20  0.04  0.07  0.51  0.10  1.03  0.12  0.72  0.81  0.50  0.75  0.57  0.29  0.01  0.93  0.70 |  |

Supplementary Table 1 (continued)

|  | 3 hours / Pre | | | 24 hours / Pre | | |
| --- | --- | --- | --- | --- | --- | --- |
|  | Fold change | -Log_10_*P* | Fold change | | -Log_10_*P* |  |
| 13-KODE  13-HpODE  16-HDoHE  17-HDoHE  9-KODE  11-HETE  13-HDoHE  10-HDoHE  8-HETE  14-HDoHE  12-HETE  11-HDoHE  7-HDoHE  8-HDoHE  5-HETE  5,6-DHET-lactone  4-HDoHE  5-KETE  Azelaoyl-PAF  AEA  OEA  EPA  DHA  AA | 0.88  17.9  0.68  0.72  1.51  0.71  0.55  0.54  0.78  0.32  1.00  0.49  1.54  0.65  0.66  0.64  0.58  1.02  0.79  0.60  0.60  0.55  0.55  0.68 | 2.05  1.07  5.08  2.55  3.00  1.96  4.93  3.03  2.67  2.68  1.80  2.39  3.54  5.61  3.50  6.57  8.31  3.22  3.80  9.39  9.31  5.56  6.93  4.62 | 1.36  23.4  1.21  2.04  3.79  2.68  1.82  2.29  1.59  5.31  5.85  3.07  2.45  1.32  1.38  1.11  1.16  4.92  1.52  0.95  1.01  1.17  1.13  1.27 | | 0.44  0.02  0.34  1.03  0.28  0.59  0.53  0.77  0.55  0.67  0.62  0.68  0.28  0.15  0.43  0.04  0.50  0.09  0.19  1.40  0.42  0.27  0.26  0.10 |  |

Abbreviations: Pre, before ICKT treatment; PG, prostaglandins; TXB_2_, thromboxane B_2_; LT, leukotrienes; DiHETE, di-hydroxy-eicosatetraenoic acids; DiHOME, dihydroxyoctadecenoic acids;

DHET, di-hydroxy-eicosatrienoic acids; HHT, hydroxy-heptadecatrienoic acid; HOTrE, hydroxy-octadecatrienoic acid; AA, arachidonic acids; HEPE, hydroxy-eicosapentaenoic acids; HETE, hydroxy-eicosatetraenoic acids; PAF, platelet-activating factors; HODE, hydroxy-octadecadienoic acids; HDoHE, hydroxyl-docosahexaenoic acids; HpODE, 13-hydroperoxy-octadecadienoic acids; KODE, oxo-octadecadienoic acids; KETE, oxo-eicosatetraenoic acids; AEA, arachidonoyl ethanolamide; EPA, eicosapentaenoic acid; DHA, docosahexaenoic acid.

-Log*P* represents -log10 (*P*-value) of paired *t*-test of Pre (before ICKT treatment) vs. 3 hours or 24 hours after ICKT treatment.Supplementary Table 2. Serum metabolites that significantly changed after 3 hours of ICKT administration.

|  | 3hr / Pre | | 24hr / Pre | |
| --- | --- | --- | --- | --- |
|  | Fold change | -Log_10_*P* | Fold change | -Log_10_*P* |
| Lactose  20-HETE  12,13-DiHOME  Fructose  Psicose  Pyruvic acid  PGD_3_*  2-Ketobutyric acid  14-HDoHE  Mannitol  Cytosine  11-HDoHE  12-HEPE  Guanosine  9,10-DiHOME  Xylose | 5.35  2.52  2.86  5.78  2.96  2.77  1177  2.03  0.31  3.59  2.99  0.48  0.46  5.07  2.51  2.03 | 6.93  5.15  4.74  4.67  4.66  4.49  4.40  2.85  2.69  2.64  2.49  2.41  2.32  2.27  2.22  2.07 | 1.77  4.22  1.01  1.27  1.22  1.45  1114  1.20  5.18  4.22  2.92  3.00  5.30  1.54  1.33  1.17 | 1.45  0.06  0.25  0.37  0.37  0.58  1.28  0.07  0.67  1.21  1.32  0.67  0.81  0.27  0.07  0.02 |

Note, 20-HETE, 20-hydroxyeicosatetraenoic acid; 12,13-DiHOME, 12,13-dihydroxy-9Z-octadecenoic acid; PGD_3_, prostaglandin D_3_; 14-HDoHE, 14(S)-hydroxy docosahexaenoic acid; 11-HDoHE, 11-hydroxy docosahexaenoic acid; 12-HEPE, 12-hydroxy-eicosapentaenoic acid; 9,10-DiHOME, 9,10-dihydroxy-12Z-octadecenoic acid. * It is revealed that the detected metabolite is not PGD_3_ by confirmation analysis using PGD_3_ standard, though it has the same MS/MS transition as that of PGD_3_.

-Log*P* represents -log10 (*P*-value) of paired *t*-test of Pre vs 3 hours or 24 hours after ICKT treatment.

Supplementary Table 3. Hepatobiliary biomarkers used for correlation analysis*

| Node name | Sample | Measurements | Time point or change** |
| --- | --- | --- | --- |
| B_T-Bil_0  B_T-Bil_24  B_T-Bil_%  B_D-Bil_0  B_D-Bil_24  B_D-Bil_%  B_BA_0  B_BA_24  B_BA_%  B_Vol_0  B_Vol_24  B_Vol_Delta  S_AST_0  S_AST_24  S_AST_%  S_ALT_0  S_ALT_24  S_ALT_%  S_T-Bil_0  S_T-Bil_24  S_T-Bil_%  S_D-Bil_0  S_D-Bil_24  S_D-Bil_%  S_ALP_0  S_ALP_24  S_ALP_%  S_GTP_0  S_GTP_24  S_GTP_% | Bile  Bile  Bile  Bile  Bile  Bile  Bile  Bile  Bile  Bile  Bile  Bile  Serum  Serum  Serum  Serum  Serum  Serum  Serum  Serum  Serum  Serum  Serum  Serum  Serum  Serum  Serum  Serum  Serum  Serum | Total bilirubin  Total bilirubin  Total bilirubin  Direct bilirubin  Direct bilirubin  Direct bilirubin  Bile acids  Bile acids  Bile acids  Bile flow  Bile flow  Bile flow  AST  AST  AST  ALT  ALT  ALT  Total bilirubin  Total bilirubin  Total bilirubin  Direct bilirubin  Direct bilirubin  Direct bilirubin  ALP  ALP  ALP  γ-GTP  γ-GTP  γ-GTP | Pre  24 hours  Change in 24 hours  Pre  24 hours  Change in 24 hours  Pre  24 hours  Change in 24 hours  Pre  24 hours  Change in 24 hours  Pre  24 hours  Change in 24 hours  Pre  24 hours  Change in 24 hours  Pre  24 hours  Change in 24 hours  Pre  24 hours  Change in 24 hours  Pre  24 hours  Change in 24 hours  Pre  24 hours  Change in 24 hours |

Pre, before ICKT treatment

* Indocyanine green retention rate at 15 minutes (ICGR15) and plasma disappearance rate of indocyanine green (ICGK) were analyzed separately because of missing values.

**For convenience, node names represented in Figure 4 and Supplementary Figures 2-4 are renamed and the new names are indicated in each figure.

**Change in 24 hours was calculated by the equation 100 × [Value in 24 hours] / [Value in Pre]. Regarding the bile volume, change in 24 hours was calculated by the equation [Value in 24 hours] - [Value in Pre]
